# Pharmacological regulation of neutrophil activity and apoptosis: Contribution to new strategy for modulation of inflammatory processes

**DOI:** 10.2478/v10102-011-0003-0

**Published:** 2011-03

**Authors:** Viera Jančinová, Tomáš Perečko, Radomír Nosáĺ, Danica Mihalová, Katarína Bauerová, Katarína Drábiková

**Affiliations:** Institute of Experimental Pharmacology & Toxicology, Slovak Academy of Sciences, SK-84104 Bratislava, Slovak Republic

**Keywords:** curcumin, neutrophils, apoptosis, caspase-3 activity, reactive oxygen species

## Abstract

Novel strategies of antiinflammatory therapy are based upon pharmacological agents capable to enhance the resolution – *i.e.* the termination of the beneficial inflammation before it may turn into an adverse chronic stage. In contrast to the current therapy, which antagonises the formation of proinflammatory mediators, the “proresolving” therapy promotes natural antiinflammatory processes. It is likely that several drugs and phytochemicals would act in this way, but this point has not been investigated and thus might be totally overlooked. In this paper, effects of curcumin (diferuloylmethane) were analysed, considering the ability of this natural compound to affect resolution of inflammation through modulation of its important inputs – activity and apoptosis of neutrophils. The presented data indicate that, besides its well-known ability to suppress mechanisms engaged at the onset and progression of inflammation, curcumin could support resolution of inflammation through decreased activity and enhanced apoptosis of neutrophils. This substance decreased the formation of oxidants in neutrophils, both under *in vitro* conditions and after oral administration to arthritic rats. Moreover, curcumin accelerated spontaneous apoptosis of neutrophils, as indicated by increased externalisation of phosphatidylserine, by intercalation of propidium iodide and by enhanced activity of the executioner caspase-3.

## Introduction

Inflammation is auniversal defense response of the organism to infection or to tissue injury. During the inflammatory process, different cytokines, growth, transforming and chemotactic factors are synthesised and several types of cells become activated. Neutrophils (polymorphonuclear leukocytes) are considered to be central cells of acute inflammation. These cells most rapidly reach the site of injury or infection and liberate antimicrobial proteins, proteases and produce reactive oxygen species. Prolonged or excessive liberation of these very effective and toxic substances could intensify the inflammatory process and enhance tissue damage. Therefore activity of neutrophils declines consecutively by the effect of antiinflammatory mediators (IL-10, lipoxins, resolvins, protectins) and programmed death of these cells (apoptosis) is initiated. These processes are essential for the termination (resolution) of the beneficial inflammation (Kohli & Levy, 2009; Serhan *et al*., 2007). An abnormal, ineffective or absent resolution contributes to tissue damage and to persisting inflammation. Moreover, it is thought to participate in the pathogenesis of chronic inflammatory disorders, such as asthma, rheumatoid arthritis or chronic obstructive pulmonary disease. Therefore novel strategies of the antiinflammatory therapy are based upon pharmacological agents capable to enhance the resolution of inflammation. In contrast to the current therapy, which antagonises the formation of proinflammatory mediators, the “proresolving” therapy promotes natural antiinflammatory processes (Filep & El Kebir, 2009; Hallett *et al*., 2008; Rossi *et al*., 2007). It is likely that several drugs and phytochemicals would act in this way, but this point has not been investigated and thus might be totally overlooked. In this paper effects of curcumin (diferuloylmethane) were analysed, considering the ability of this natural compound to affect resolution of inflammation through modulation of its important inputs – activity and apoptosis of neutrophils.

## Materials and methods

### Chemicals

The highly purified curcumin (diferuloylmethane), luminol, isoluminol and PMA (phorbol-myristate-acetate) were obtained from Sigma (Steinheim, Germany) and methotrexate was from Pliva-Lachema (Brno, Czech Republic). Human Annexin V/FITC Kit was purchased from Bender MedSystems (Vienna, Austria), Caspase-Glo 3/7 Assay from Promega (Madison, Wis, USA) and human purified caspase-3 was from Enzo Life Sciences (Lausen, Switzerland).

### Neutrophil activity

Activity of neutrophils was evaluated on the basis of oxidant formation measured by the chemiluminescence method. Under *in vitro* conditions, isolated human neutrophils were stimulated with 0.05 µM PMA and chemiluminescence was enhanced either with isoluminol (extracellular) or with luminol in the presence of extracellular scavengers – superoxide dismutase and catalase (Nosál *et al*., 2009). The effect of orally administered curcumin on neutrophil activity was assessed in arthritic rats, in compliance with Principles of Laboratory Animal Care (Bauerová *et al*., 2009). The study was approved by the State Veterinary and Food Administration of the Slovak Republic and by the Ethical Committee at the Institute of Experimental Pharmacology and Toxicology. Curcumin (50 mg/kg, daily) or methotrexate (0.5 mg/kg, twice a week) was administered orally over a period of 28 days after arthritis induction. Then the number of neutrophils and the luminol enhanced whole blood chemiluminescence (spontaneous or stimulated with 0.01 µM PMA) were assessed (Drábiková *et al*., 2009). The presented data, expressed in RLU (relative light units), are based on integral values of chemiluminescence over 1800 s (isolated neutrophils) or 3600 s (whole blood).

### Neutrophil apoptosis

Viability of human neutrophils was recorded by flow cytometry after double staining with Annexin-V conjugated with FITC (fluorescein isothiocyanate) and propidium iodide. Only Annexin positive cells were considered pre-apoptotic cells and double positive cells (Annexin+/propidium+) were considered late-apoptotic or dead cells.

### Caspase-3 activity

Effect of curcumin on caspase-3 activity was evaluated in cell-free system by the luminescence method, using Caspase-Glo 3/7 Assay kit and human purified caspase-3 (Perečko *et al*., 2010). The method is based on caspase cleavage of Z-DEVD-amino-luciferin substrate and on the light production resulting from the reaction of amino-luciferin with luciferase.

### Statistical analysis

All values are given as means±SEM. The statistical significance of differences between means was established by Student's t-test and *p*-values below 0.05 were considered statistically significant.

## Results

Under *in vitro* conditions, curcumin decreased the concentration of oxidants, produced by activated neutrophils both intra- and extracellularly. However its concentration needed for a significant modulation of the radicals formed inside neutrophils was 100-times higher than that effective extracellularly (Table 1). The effect of curcumin on neutrophil activity was further tested under *in vivo* conditions – in arthritic rats. Adjuvant arthritis was accompanied by an increased number of neutrophils in blood and by a more pronounced spontaneous and stimulated chemiluminescence (Table 2). Whereas the arthritis related alteration in neutrophil count and in spontaneous chemiluminescence were not modified by curcumin, the increased reactivity of neutrophils to PMA was less evident in curcumin treated animals. The effects of curcumin were similar to those of methotrexate – a drug widely used in the therapy of arthritic patients.

**Table 1:** Effect of curcumin on neutrophil activity *in vitro*.

	Percentage of inhibition	
	
Curcumin (µM)	Intracellular chemiluminescence	Extracellular chemiluminescence
0.01	8.18±4.56	9.92±3.82
0.1	6.93±5.51	15.66±3.84**
1	8.09±6.07	31.03±1.85**
10	81.40±2.43**	86.09±2.30**
100	93.88±1.38**	99.70±0.04**

Formation of reactive oxygen species was measured on the basis of luminol/isoluminol enhanced chemiluminescence in human neutrophils treated with curcumin and stimulated with PMA. Absolute control values of chemiluminescence, expressed in relative light units, were 465200±75789 RLU (intracellular) and 9487833±1125 207 RLU (extracellular). Mean±SEM, n=6, ** *p<*0.01

**Table 2:** Effect of curcumin on neutrophil activity in arthritis.

	Chemiluminescence (RLU)		
		
	Spontaneous	PMA stimulated	Number of neutrophils in 1 µl of blood
**Control**	54777±3170	60412±2557	8583±802
**Arthritis**	84008±7841^++^	543700±61389^++^	22883±1240^++^
**Arthritis+curcumin**	107898±17992	187867±26611**	28883±5865
**Arthritis+methotrexate**	89854±15661	128200±18435**	19540±3326

Spontaneous and PMA stimulated whole blood chemiluminescence and neutrophil count measured in healthy (control) and not medicated arthritic rats as well as in arthritic animals treated with curcumin (50 mg/kg, daily) or with methotrexate (0.5 mg/kg, twice a week). Mean±SEM, n=6, ^++^*p<*0.01 (vs Control), **p<0.01 (vs Arthritis), RLU – relative light units.

**Figure 1:**
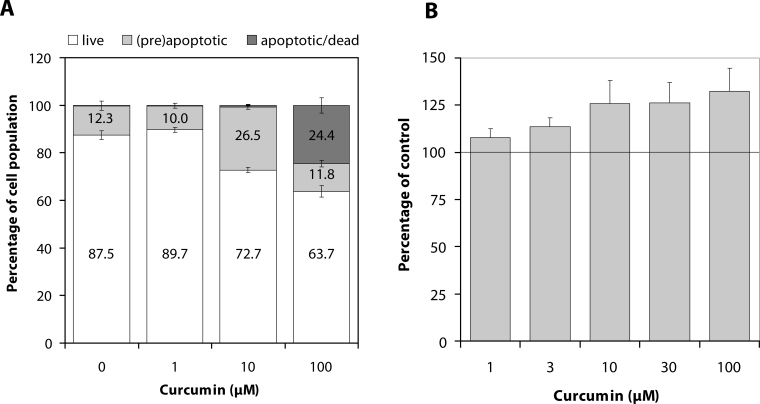
Neutrophil apoptosis (**A**) and caspase-3 activity (**B**) in the presence of curcumin. The life-span of human neutrophils was recorded by flow cytometry, using double staining with Annexin-V (AN) and propidium iodide (PI). Of the population of 5000 granulocytes, the percentage of live (AN^−^/PI^−^), preapoptotic (AN^+^/PI^−^) and apoptotic (AN^+^/PI^+^) cells was calculated. The activity of human recombinant caspase-3 was measured luminometrically, by assessing cleavage of a tagged peptide substrate. Mean±SEM, n=5–6.

Curcumin (10 and 100 µM) elevated the percentage of preapoptotic and apoptotic neutrophils (Figure 1A) and increased the activity of human recombinant caspase-3 (Figure 1B).

## Discussion

Curcumin is anatural antioxidant, displaying remarkable antiinflammatory and antiarthritic activities (Funk *et al*., 2006; Jagetia & Aggarwal, 2007). These beneficial effects have been attributed to the capacity of curcumin to prevent activation of nuclear factor-kappa B and the subsequent overexpression of proinflammatory mediators – cytokines, adhesion molecules, cyclooxygenase-2, phospholipase A_2_, myeloperoxidase, collagenase, as well as to its ability to modulate activities of T lymphocytes and macrophages (Huang *et al*., 2004; Sharma *et al*., 2005). The presented results indicate that, besides suppression of proinflammatory processes, curcumin could promote resolution of inflammation owing to its capacity to accelerate apoptosis of neutrophils and to inhibit oxidative burst of these cells.

As shown by flow cytometric analysis, curcumin enhanced spontaneous apoptosis of neutrophils. This was indicated by an increased number of Annexin positive cells – *i.e.* cells displaying a more pronounced externalisation of phosphatidylserine. The expression of phosphatidylserine on the external side of plasma membrane facilitates the recognition of apoptotic neutrophils by macrophages and their safe removal from the site of inflammation (Luo & Loison, 2007). At high concentration (100 µM), curcumin increased the number of late apoptotic or dead neutrophils, disclosed on the basis of nucleus staining by propidium iodide. Integrity of these cells was not destroyed by curcumin treatment and thus the discharge of toxic neutrophil contents into the extracellular space was prevented.

The proapoptotic effect was not confirmed under *in vivo* conditions – by a decreased number of neutrophils in rats treated with curcumin. This might be due to the lower oral bioavailability of curcumin (Aggarwal & Sung, 2008) and to the dosage/period of administration – not sufficient to achieve effective concentration in neutrophils.

The ability of curcumin to increase apoptosis has been extensively studied in cancer cells (Nair *et al*., 2010). In neutrophils, it was deduced from the altered morphology and from the activation of the executioner caspase-3 in these cells, resulting from the activation of p38 mitogen-activated protein kinase (Hu *et al*., 2005). Our experiments, performed in cell-free system, indicated that direct interaction with caspase-3, resulting in increased activity of this enzyme, could not be excluded.

Reduced formation of reactive oxygen species in neutrophils may represent another mechanism involved in the proresolving activity of curcumin. Oxidants released outside neutrophils (potentially dangerous for host tissues) were reduced more effectively than oxidants formed within neutrophils (involved in elimination of phagocytosed pathogens and fulfilling a regulatory role). Compared to previous studies (Limasset *et al*., 1993; Jackson *et al*., 2006), the inhibition was observed at a lower concentration of curcumin. Reduced chemiluminescence could result from the well-known scavenging activity of curcumin, which originates from the presence of phenolic and central methylene hydrogens in its molecule (Weber *et al*., 2005; Pari & Murugan, 2006). Moreover, curcumin was found to decrease phosphorylation of the signalling enzyme protein kinase C (PKC), namely of its two isoforms PKCα and PKCβII (Jančinová *et al*., 2009). Since these isoforms directly participate in the activation of the neutrophil NADPH oxidase (Fontayne *et al*., 2002), their inhibition could result in reduced oxidant formation and may explain the decreased chemiluminescence observed in the presence of curcumin.

The decreasing effect of curcumin on neutrophil activity was further observed under *in vivo* conditions – in arthritic rats. Neutrophils modified by inflammation produced several times more oxidants than did healthy controls and this hyperreactivity was suppressed by orally administered curcumin. Since the excessive formation of radicals was ascribed to the enhanced activity of NADPH oxidase in the presence of cytokines (Fairhurst *et al*., 2007; Miesel *et al*., 1996), the capacity of curcumin to prevent activation of nuclear factor-kappa B and the subsequent expression of proinflammatory mediators (Funk *et al*., 2006; Huang *et al*., 2004; Jagetia & Aggarwal, 2007; Sharma *et al*., 2005) could be involved in this effect.

Curcumin-related inhibition of neutrophil oxidative burst was comparable with the activity of methotrexate – a drug widely used in the therapy of arthritic patients, which triggers synthesis of the endogenous anti-inflammatory mediator adenosine (Cronstein, 2005) and is thus considered a “resolution friendly” drug (Serhan *et al*., 2007).

The presented data indicate that, besides its ability to suppress mechanisms engaged at the onset and progression of inflammation, curcumin could support resolution of inflammation through decreased activity and enhanced apoptosis of neutrophils.
